# The Prognostic Impact of Molecular Subtypes and Very Young Age on Breast Conserving Surgery in Early Stage Breast Cancer

**DOI:** 10.7759/cureus.633

**Published:** 2016-06-07

**Authors:** Cetin Ordu, Kandace McGuire, Gul Alco, Kezban Nur Pilanci, Ulkuhan I Koksal, Filiz Elbüken, Zeynep Erdogan, Filiz Agacayak, Serkan Ilgun, Dauren Sarsenov, Alper Öztürk, Şefik İğdem, Sait Okkan, Yeşim Eralp, Maktav Dincer, Vahit Ozmen

**Affiliations:** 1 Medical Oncology, Gayrettepe Florence Nightingale Hospital; 2 General Surgery, University of North Carolina at Chapel Hill; 3 Radiation Oncology, Gayrettepe Florence Nightingale Hospital; 4 Medical Oncology, Istanbul Bilim University; 5 Radiology, Gayrettepe Florence Nightingale Hospital; 6 Physical Therapy and Rehabilitation, Istanbul Bilim University; 7 Radiology, Istanbul Florence Nightingale Hospital; 8 General Surgery, Istanbul Florence Nightingale Hospital; 9 Medical Oncology, Istanbul University/Oncology Institute; 10 Department of Surgery, Istanbul University

**Keywords:** young age, breast cancer, survival, breast conserving surgery, molecular subtypes

## Abstract

**Background:**

Premenopausal breast cancer with a triple-negative phenotype (TNBC) has been associated with inferior locoregional recurrence free survival (LRFS) and overall survival (OS) after breast conserving surgery (BCS). The aim of this study is to analyze the association between age, subtype, and surgical treatment on survival in young women (≤40 years) with early breast cancer in a population with a high rate of breast cancer in young women.

**Methods:**

Three hundred thirty-two patients ≤40 years old with stage I-II invasive breast cancer who underwent surgery at a single institution between 1998 and 2012 were identified retrospectively. Uni- and multivariate analysis evaluated predictors of LRFS, OS, and disease free survival (DFS).

**Results:**

Most patients (64.2%) underwent BCS. Mean age and follow-up time were 35 (25 ± 3.61) years, and 72 months (range, 24–252), respectively. In multivariate analysis, multicentricity/multifocality and young age (<35 years) independently predicted for poorer DFS and OS. Those aged 35–40 years had higher LRFS and DFS than those <35 in the mastectomy group (p=0.007 and p=0.039, respectively). Patients with TNBC had lower OS compared with patients with luminal A subtype (p=0.042), and those who underwent BCS had higher OS than patients after mastectomy (p=0.015).

**Conclusion:**

Young age (< 35 years) is an independent predictor of poorer OS and DFS as compared with ages 35–40, even in countries with a lower average age of breast cancer presentation. In addition, TNBC in the young predicts for poorer OS. BCS can be performed in young patients with TNBC, despite their poorer overall survival.

## Introduction

Breast cancer (BC) is one of the most common cancers and a leading cause of cancer-related death among women worldwide. Although the incidence of BC in developed countries is higher, frequency and mortality rates have increased in the past decades in developing countries, because of decreased infant mortality and other causes of early death [1] Due to the older populations in developed countries, only 25% of patients with BC are premenopausal (and/or <50 years of age) and only 5–6% of them are younger than 40 years of age at presentation [2]. Conversely, BC incidence in premenopausal women, especially the very young (< age 40), are higher in low-middle income countries as a result of young age structure [2]. The burden of BC in Turkey has doubled in the last two decades. Almost half of all BC patients are premenopausal at presentation and 20% are younger than 40 years old. Women <40 years of age present with advanced stage disease and have an overall poorer prognosis than their older counterparts. There is a mammographic screening program in Turkey, but women under the age of 40 do not benefit from screening, as studies from more developed nations show, despite the higher incidence of BC in this population. Turkey is fortunate to have a national cancer registry and a well-developed breast cancer database that was begun in 2005 and now includes more than 20,000 patients [2-3]. 

BC in young women is more likely to involve large, lymph node-positive tumors that exhibit lymphovascular invasion, higher histologic grade, and hormone receptor negativity at diagnosis. Younger women have an increased risk of recurrence and death from BC compared with older women [4-5]. These variations may be explained by biologic differences, although delay in diagnosis may also contribute to presentation with more advanced stage disease and, thus, have an impact on prognosis. Early studies have established a continuous linear effect, with a four percent decrease in distant recurrence and six percent in local recurrence for every additional year of age [6]. And while some early papers described age as an independent risk factor for OS, more recent studies suggest that age is simply a surrogate risk factor that correlates strongly with other poor prognostic factors [7]. Numerous studies have evaluated clinico-pathological and treatment-related factors, including age, menopausal status, nodal status, margin status, presence of lymphovascular invasion, receptor status, and human epidermal growth factor (HER2) gene expression, that may increase risk of local recurrence [8]. Young age as an independent predictive factor for local recurrence after BCS remains a controversial topic. Whereas several studies have reported higher local recurrence rates in young women [9-10], other studies have reported no difference between young and older age groups [11-12]. Factors that impact the results of these studies include small sample sizes, differing definitions of young age, and tumor characteristics specific to younger women.

While some preliminary studies suggested that the distribution of histologic subtypes is different in young women with a higher prevalence of triple negative and HER2+ disease, a clear molecular characterization of BC in these patients is lacking [13-14]. Though triple negative breast cancer (TNBC) is being associated with poorer OS, it is not related with increased risk for locoregional relapse after conservative surgery. So, TNBC can be managed with better survival outcomes using conservative surgery than aggresive surgery [15-16].

The purpose of this study is to analyze the association between prognostic factors, molecular subtypes, and surgical treatment on overall and disease-free survival in young women (≤40 years) with early breast cancer, in a large cohort of young women in a developing country.

## Materials and methods

Patients’ data was identified retrospectively from the archives of Florence Nightingale Breast Study Group, Istanbul, between January 1998 and January 2012. All patients provided informed consent for their information to be stored in the hospital database and be used for research. Patients who were treated either with mastectomy or BCS and were ≤40 years old at the time of diagnosis, with clinically early stage BC (Stage I or II) as determined by physical examination and screening methods (mammography and breast and axillary ultrasound) were included. Patients were excluded if they received neoadjuvant chemotherapy, had bilateral breast cancer or had less than two years of follow-up. Analyzes were done according to pathological characteristics such as pathological stage, lymphovascular invasion (LVI), histological grading (modified Scarff-Bloom-Richardson grading), presence of in situ carcinoma, multicentricity (tumor in more than one quadrant)/ multifocality (multiple tumors in a single quadrant) (MC/MF), Ki 67%, immunohistochemical receptor status (estrogen receptor (ER), progesterone receptor (PR)), and human epidermal growth factor (HER2). If HER2 was uncertain by immunohistochemistry, either fluorescence in situ hybridization (FISH) or chromogenic in situ hybridization (CISH) were performed. Also, the patients’ demographic features, adjuvant treatments, and molecular subtypes were recorded.

### Statistics

Overall survival (OS) time was calculated from the date of surgery to the date of breast cancer specific death or the last follow-up. Local recurrence free survival (LRFS) was calculated from the date of surgery to the date of local recurrence(LR). We used the term cumulative incidence to specify the occurrence as a percentage of local recurrence at well-defined follow-up points. Likewise, we used the term survival to specify the percentage of patients still alive, and thus at risk, at well-defined follow-up points. The crude probability of death or LR was estimated by using the Kaplan-Meier method and differences between patient groups were assessed by the log-rank test. Estimated relative risks of death or LR were expressed as hazard ratios (HR) and their corresponding 95% confidence intervals (95% CI). Univariate Cox regression models were used to evaluate the effect of each specific parameter. Multivariate Cox regression models with stepwise selection were performed to identify the major significant death or LR occurrence predictors. All patients are included in all analyses. The statistical results were considered significant at a p value < 0.05. All statistical tests were performed using SPSS 17 software (IBM Corporation, New York, USA).

## Results

We identified 1550 patients with BC, 332 of whom (20.7%) were young (≤40 years). Among these young patients, 39% were under 35 years of age. The BCS rate in young patients was 64.2%. The median follow-up time was 72 months (range, 24–252 months) and the mean age was 35 years (25 ± 3.61) (Table 1).

**Table 1:** Patient and Tumor Characteristics by Age Group *p<0.01; a=Chi-Square Test; Mann-Whitney U Test, MC/MF - Multicentricity/multifocality, IDC - Invasive ductal carcinoma, LVI - Lymphovascular invasion.

	Age <35 (n=125)		Age 35–40 (n=207)				
	n	%	n	%	Total		p^a^
Breast surgery type							
BCS	82	65.6	131	63.3	213	64.2	0.670
Mastectomy	43	34.4	76	36.7	119	35.8	
pT Stage							
1	66	52.8	107	51.7	173	52.1	0.845
2-3	59	47.2	100	48.3	159	47.9	
pN Stage							
pN0	61	48.8	109	52.7	170	51.2	0.496
pN1-3	64	51.2	98	47.3	162	48.8	
p Stage (TNM)							
1	42	33.6	72	34.8	114	34.3	0.826
2-3	83	66.4	135	65.2	218	65.7	
Histology							
IDC	110	88.0	179	86.5	289	87.0	0.688
Others	15	12.0	28	13.5	43	13.0	
Tumor focality							
Unifocal	103	82.4	142	68.6	245	73.8	0.006*
MC/MF	22	17.6	65	31.4	87	26.2	
Histological grade							
I-II	58	46.4	110	53.1	168	50.6	0.234
III	67	53.6	97	46.9	164	49.4	
LVI							
Positive	67	54.5	110	53.4	177	53.8	0.850
Negative	56	45.5	96	46.6	152	46.2	
In situ component (n=328)							
Positive	87	70.7	145	70.7	232	70.7	1.000
Negative	36	29.3	60	29.3	96	29.3	
ER							
Negative	36	28.8	53	25.6	89	26.8	0.524
Positive	89	71.2	154	74.4	243	73.2	
PR							
Negative	86	68.8	145	70.0	231	69.6	0.811
Positive	39	31.2	62	30.0	101	30.4	
Her-2 (n=328)							
Negative	94	76.4	143	69.8	237	72.3	0.192
Positive	29	23.6	62	30.2	91	27.7	
Chemotherapy							
Received	116	92.8	178	86.0	294	88.6	0.087
Non- Received	9	7.2	29	14.0	38	11.4	
Hormonal therapy							
Received	95	76.0	161	77.8	256	77.1	0.709
Non-Received	30	24.0	46	22.2	76	22.9	
Median Tm diameter	20	(2-110)	20	(1-110)	20	(1-110)	0.494
Median Ki-67 %	25	(5-85)	30	(2-95)	30	(2-95)	0.390

Most of patients (87%) had invasive ductal carcinoma (IDC), others had invasive lobular carcinoma (ILC) (3.6%), mixed type (3.6%), and metaplastic cancer (5.3%). During follow-up, there were 19 (5.9%) breast cancer specific deaths and 21 (6.3%) locoregional recurrence rate (LRR). Tumor size, pathologic stage, multicentric/multifocal (MC/MF) disease, lymphovascular invasion (LVI), extensive intra-ductal component (EIC), and the receipt of systemic therapy were significantly higher in the mastectomy group (Table 1). Patients were classified into four molecular subtypes based on the latest St. Gallen Consensus Conference [17]. The molecular subtype distribution was as follows: luminal A (42%), luminal B (34%), HER2 positive (9%), and triple negative (13.5%). Patients younger than 35 years have a nonsignificantly lower incidence of luminal A subtype and higher triple negative subtype when compared with patients aged 35–40 years [46 (36 %) vs 107 (51%) and 25 (20%) vs 27 (13%)]. LRFS, DFS, and OS rates for patients ≤40 years of age were 95%, 83%, and 95% respectively at five years.

### Predictors of disease free and overall survival

**Figure 1:**
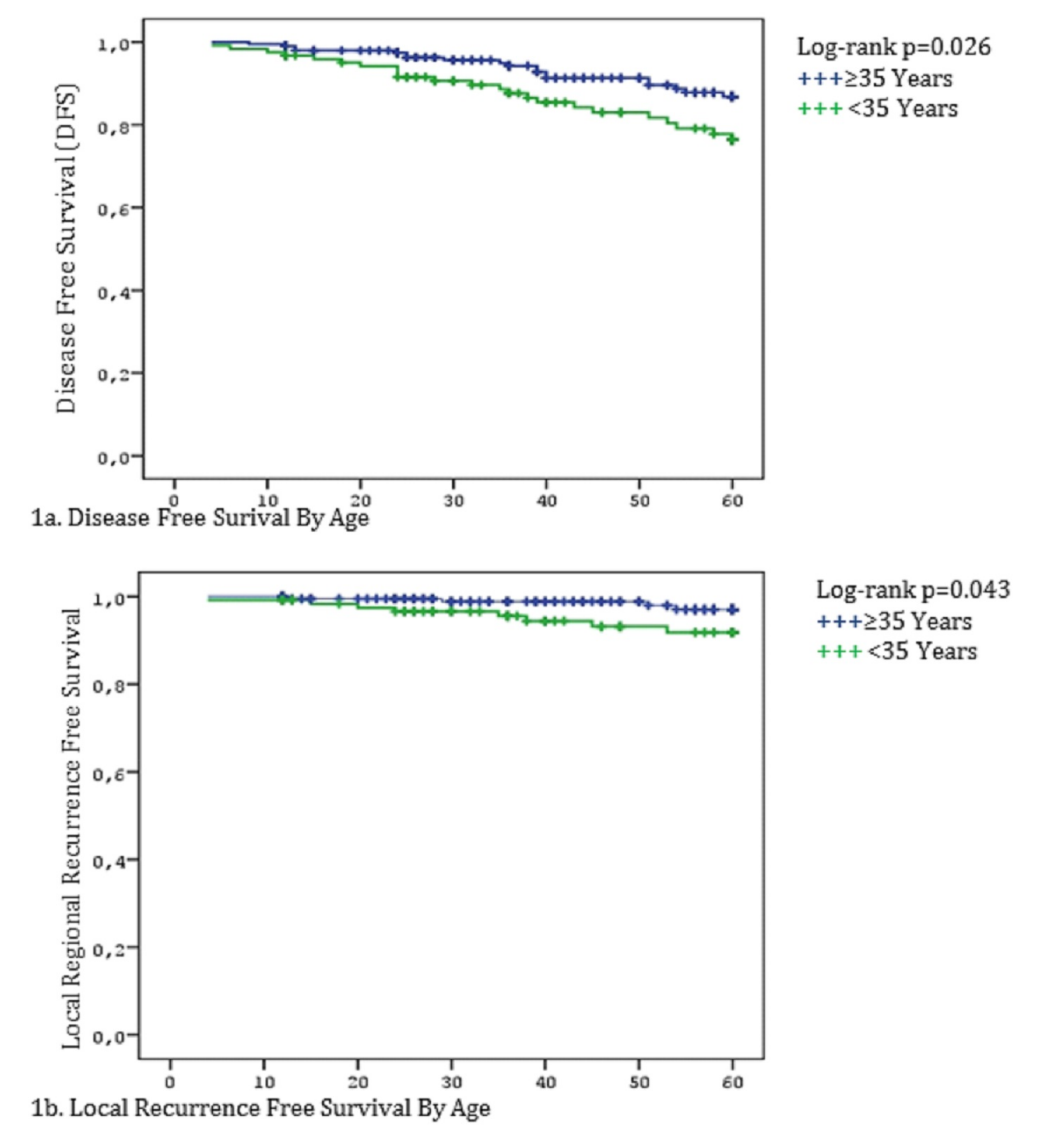
Breast Cancer Outcomes by Age Younger patients had lower LRFS and DFS rates than the older age group (<35 years vs 35–40 years).

**Figure 2:**
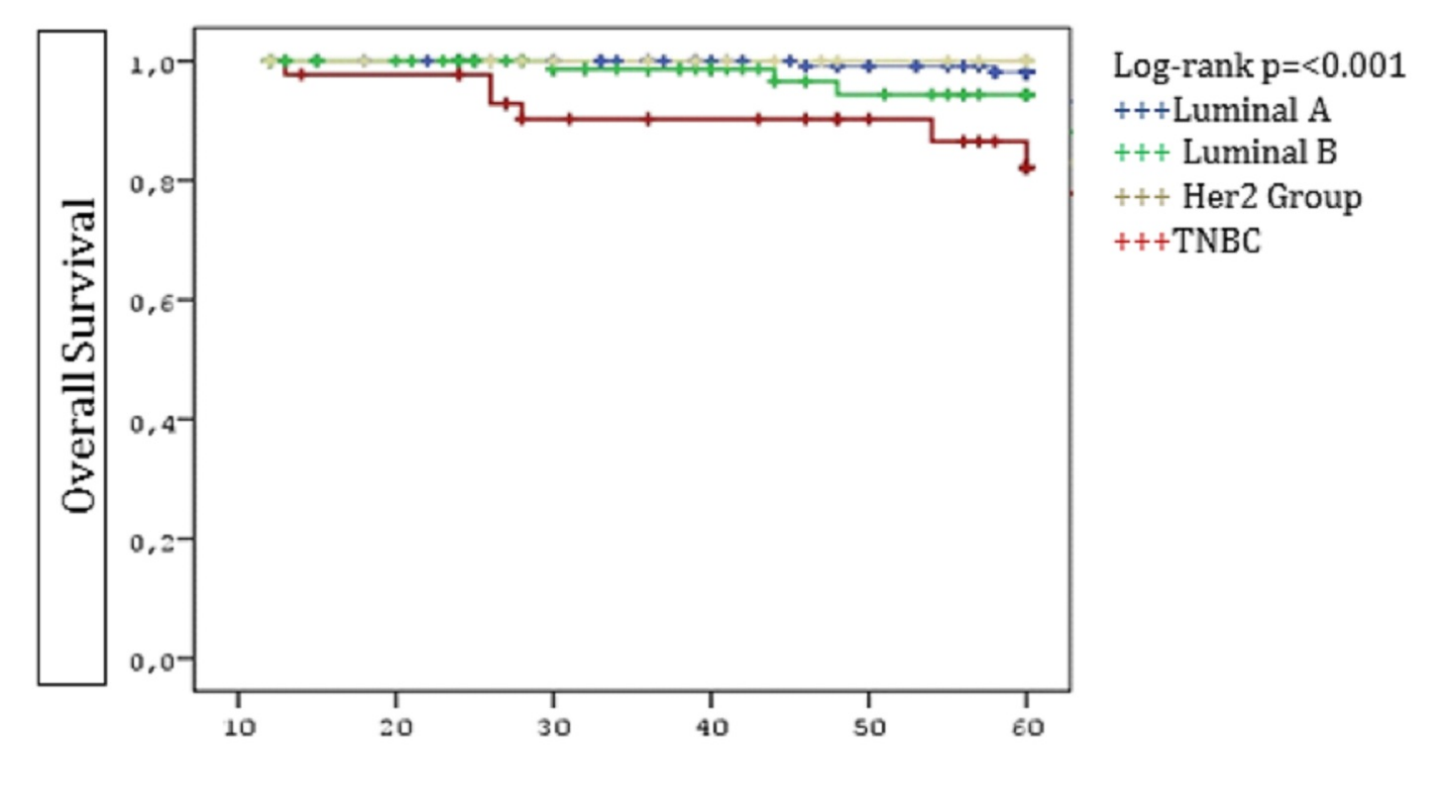
Overall Survival by Molecular Subtypes Triple negative breast cancer patients (TNBC) had significantly lower OS than luminal-A subtype.

In univariate analysis, advanced pathologic stage (II-III), LVI, MC/MF, and younger age (<35 years) were associated with poorer DFS. Advanced pathologic stage (II-III), MC/MF, hormone receptor negativity (ER, PR), LRR, and distant metastasis were associated with decreased OS. In multivariate analysis, MC/MF and younger age were independent predictors for both decreased DFS and OS (Table 2). Additionally, advanced pN stage (pN2-3), and PR negativity (-) were independent predictors of worse OS. When comparing patients by age (<35 years vs 35–40 years), younger patients had lower LRFS and DFS rates than the older age group (p=0.043 and p=0.026, respectively, as seen in Table 2, Figure 1). TNBC had significantly lower OS than luminal-A subtype (p=0.042, Figure 2).

**Table 2:** Multivariate Analysis with Cox Model of Predictors for Disease Free and Overall Survival DFS - Disease free survival OS - Overall survival HR - Hazard ratio PR - Progesterone receptor pN - Pathological node CI - Confidence interval

	Variables	HR (95%CI)	p value
DFS at 5 years	Age (<35 year)	2.375 (1.263-4.465)	0.007
	Multicentricity/Multifocality	2.802 (1.485-5.284)	0.001
OS at 5 years	Age (<35 year)	3.859 (1.244-11.967)	0.019
	Multicentricity/Multifocality	3.942 (1.378-11.273)	0.011
	PR (Negative)	4.946 (1.704-14.355)	0.003
	pN Stage (pN+)	3.891 (1.213-12.480)	0.022

### Local therapy, age, and survival

We also analyzed the effect of local therapy on LRFS, DFS, and OS, as stratifed by age. Age <35 years was associated with lower LRFS and DFS after mastectomy when compared to age 35–40 years (p=0.007, and p=0.039, respectively). However, these differences were not statistically significant in patients who underwent BCS. In addition, age <35 years was associated with a worse OS after BCS, but not after mastectomy (Table 3).

**Table 3:** Survival Association Between Age and Surgery BCS - Breast conserving surgery LRFS - Local recurrence free survival DFS - Disease free survival OS - Overall survival * Mann Whitney-U Test

	LRFS (% at 5 years)		
Type of Surgery	< 35 years	35-40 years	p value
BCS	94.0	95.2	0.565
Mastectomy	87.5	100.0	0.007
p value	0.349	0.115	
	DFS (% at 5 years)		
Type of Surgery	<35 years	35-40 years	p value
BCS	82.7	88.4	0.223
Mastectomy	68.6	84.2	0.039
p value	0.059	0.376	
	OS (% at 5 years)		
Type of Surgery	<35 years	35-40 years	p value
BCS	93.3	97.9	0.018
Mastectomy	92.3	93.7	0.717
p value	0.627	0.015*	

## Discussion

Young age is an established risk factor for poorer prognosis after the diagnosis of breast cancer. Our trial revealed that breast cancer in patients younger than 35 years (as opposed to 35–40) was an independent risk factor for poorer OS. Despite the fact that some of published trials have defined young age under 35 or 40 years as a risk factor for survival in early breast cancer, defining certain thresholds of age are controversial. Anders, et al. found that age under 40 years, conferred an inferior DFS when compared with ages of 40–45 years at breast cancer diagnosis. [13]. Some trials refer to age <35 years, as a risk factor for LR and poor OS [18]. A study from Korea that included patients who were under 30 years and 30–34 years old at the time of diagnosis, showed that the risk of death rose by five percent for every one-year reduction in age, even among patients <34 years [19]. Because ovarian function decreases suddenly around the age of 37 years, identifying a threshold for young age between 35 and 40 years may be rational. It is plausible that the changes that occur during the peak of the reproductive years (12–14 years before menopause) may play a role in the biology of tumors arising in this period [20].

Meta-analysis of multiple randomized trials have demonstrated that BCS provides survival equivalent to that seen with mastectomy for patients with early-stage breast cancer [21]. But, BCS in young patients is a controversial issue. Several retrospective trials showed that BCS in young patients was associated with increased LRR [18-24]. Some studies have also suggested that young women (less than 35 or 40 years of age) have inferior cosmetic outcomes with BCS, implying that such women may be better served by mastectomy [22]. However, there are trials indicating that breast cancers in young women can be managed safely with BCS with similar OS and LRR as that in older patients [23-24]. In a meta-analysis comparing BCS and mastectomy in young patients, no difference was found in LRR and OS rates [25]. In this study, there were no differences in LRFS, DFS, and OS rates at five years between mastectomy and BCS in young patients (≤ 40 years). Whereas the LRR rate in previous trials was between 6–24%, it was 6.3% in our study.

Overall survival is the ultimate goal in cancer therapy and our study addresses OS in this cohort of young patients. Bantema-Joppe, et al. reported that patients who were ≤40 years old had higher LRR after BCS versus mastectomy, but at median nine years of follow-up, OS in the BCS group was significantly higher than the mastectomy group [28]. Similar to Bantema-Joppes’s study, our study revealed that patients treated with mastectomy had more advanced stage disease (e.g. tumor size, pathologic stage, MC/MF disease, LVI, EIC). When we divided patients into two age categories (<35 vs 35–40 years), we found that the LRFS and DFS rates were significantly lower in younger patients who had mastectomy. Other published trials have similarly shown that younger age in breast cancer patients treated with mastectomy is associated with poor prognosis [6, 27]. Many trials have demonstrated that breast cancer in young women (<40 years) can be safely managed with BCS [24-25]. Based on the U.S. Surveillance, Epidemiology and End Results (SEER) database, Mahmood, et al. reported that there were no differences in outcomes for local treatment when stratified by age quartile [26]. Explanations for this are suggested to be multifactorial, including a more careful evaluation of tumor margins, more extensive and accurate use of boost radiation to the tumor bed, and more patients receiving adjuvant systemic therapy.

Despite the body of evidence that suggests that patients ≤40 years of age with breast cancer can be managed with BCS, the question remains whether young breast cancer patients <35 years old can be treated with BCS appropriately. The results of our study and those of others suggest that young age is a prognostic, rather than predictive factor, and that young age by itself is not a contraindication for BCS [23, 26].

Although some studies did show the presence of MC/MF tumors as an independent risk factor for both OS and progression free survival, it was an independent risk factor in our study [29]. Axillary lymph node positivity and triple negative tumor phenotype were also independent risk factors for OS, consistent with the literature [30]. Thus it appears that mastectomy itself is not a predictor of poor outcome, so much as it is a marker of more advanced/biologically aggressive disease.

Despite being based on retrospective data, the strength of our current trial is its ability to analyze breast cancer in young patients from a relatively homogeneous population. Also, the study period between the dates of 1998 and 2012 is more recent compared with other trials in the literature. In fact, 80% of patients who underwent adjuvant chemotherapy received either a taxane or trastuzumab after 2005. This may explain the low LRR and better survival rates in our study as compared to other trials in similar age groups.

## Conclusions

This study showed that among young patients (≤40 years) with breast cancer, very young age (<35 years) at presentation is an independent risk factor for decreased OS and DFS. In addition, it is associated with lower OS in patients who are treated with BCS. Young age (<35 years) is associated with lower DFS and LRFS in patients who are treated with mastectomy. We suggest that age <35 years is a risk factor for poorer overall survival regardless of surgical therapy.

## References

[1] Parkin DM, Bray FI, Devesa SS (2001). Cancer burden in the year 2000. The global picture. Eur J Cancer.

[2] Brinton LA, Sherman ME, Carreon JD, Anderson WF (2008). Recent trends in breast cancer among younger women in the United States. J Natl Cancer Inst.

[3] Vahit Ozmen (2013). Breast Cancer in Turkey. Breast.

[4] Fredholm H, Eaker S, Frisell J, Holmberg L, Fredriksson I, Lindman H (2009). Breast cancer in young women: poor survival despite intensive treatment. PLoS One.

[5] Han W, Kim SW, Park IA, Kang D, Kim SW, Youn YK, Oh SK, Choe KJ, Noh DY (2004). Young age: an independent risk factor for disease-free survival in women with operable breast cancer. BMC Cancer.

[6] de la Rochefordiere A, Campana F, Fenton J, Vilcoq JR, Fourquet A, Asselain B, Scholl SM, Pouillart P, Durand JC, Magdelenat H (1993). Age as prognostic factor in premenopausal breast carcinoma. Lancet.

[7] Partridge AH, Gelber S, Piccart-Gebhart MJ, Focant F, Scullion M, Holmes E, Winer EP, Gelber RD (2013). Effect of age on breast cancer outcomes in women with human epidermal growth factor receptor 2-positive breast cancer: results from a herceptin adjuvant trial. J Clin Oncol.

[8] Arvold ND, Taghian AG, Niemierko A, Abi Raad RF, Sreedhara M, Nguyen PL, Bellon JR, Wong JS, Smith BL, Harris JR (2011). Age, breast cancer subtype approximation, and local recurrence after breast-conserving therapy. J Clin Oncol.

[9] Miles RC, Gullerud RE, Lohse CM, Jakub JW, Degnim AC, Boughey JC (2012). Local recurrence after breast-conserving surgery: multivariable analysis of risk factors and the impact of young age. Ann Surg Oncol.

[10] Gentilini O, Botteri E, Rotmensz N, Toesca A, De Oliveira H, Sangalli C, Colleoni M, Intra M, Galimberti V, Veronesi P, Luini A, Veronesi U (2010). Breast-conserving surgery in 201 very young patients (<35 years). Breast.

[11] Rapiti E, Fioretta G, Verkooijen HM, Vlastos G, Schäfer P, Sappino AP, Kurtz J, Neyroud-Caspar I, Bouchardy C (2005). Survival of young and older breast cancer patients in Geneva from 1990 to 2001. Eur J Cancer.

[12] Chia KS, Du WB, Sankaranarayanan R, Sankila R, Wang H, Lee J, Seow A, Lee HP (2004). Do younger female breast cancer patients have a poorer prognosis? Results from a population-based survival analysis. Int J Cancer.

[13] Anders CK, Hsu DS, Broadwater G, Acharya CR, Foekens JA, Zhang Y, Wang Y, Marcom PK, Marks JR, Febbo PG, Nevins JR, Potti A, Blackwell KL (2008). Young age at diagnosis correlates with worse prognosis and defines a subset of breast cancers with shared patterns of gene expression. J Clin Oncol.

[14] Anders CK, Fan C, Parker JS, Carey LA, Blackwell KL, Klauber-DeMore N, Perou CM (2011). Breast carcinomas arising at a young age: unique biology or a surrogate for aggressive intrinsic subtypes?. J Clin Oncol.

[15] Pogoda K, Niwińska A, Murawska M, Pieńkowski T (2013). Analysis of pattern, time and risk factors influencing recurrence in triple-negative breast cancer patients. Med Oncol.

[16] Rakha EA, Reis-Filho JS, Ellis IO (2008). Basal-like breast cancer: a critical review. J Clin Oncol.

[17] Goldhirsch A, Winer EP, Coates AS, Gelber RD, Piccart-Gebhart M, Thürlimann B, Senn HJ, panel members (2013). Personalizing the treatment of women with early breast cancer: highlights of the St Gallen International Expert Consensus on the Primary Therapy of Early Breast Cancer 2013. Ann Oncol.

[18] Peng R, Wang S, Shi Y, Liu D, Teng X, Qin T, Zeng Y, Yuan Z (2011). Patients 35 years old or younger with operable breast cancer are more at risk for relapse and survival: a retrospective matched case-control study. Breast.

[19] Kim EK, Noh WC, Han W, Noh DY (2011). Prognostic significance of young age (<35 years) by subtype based on ER, PR, and HER2 status in breast cancer: a nationwide registry-based study. World J Surg.

[20] Azim HA Jr, Azim H (2013). Breast cancer arising at a young age: do we need to define a cut-off?. Breast.

[21] Clarke M, Collins R, Darby S, Davies C, Elphinstone P, Evans V, Godwin J, Gray R, Hicks C, James S, MacKinnon E, McGale P, McHugh T, Peto R, Taylor C, Wang Y; Early Breast Cancer Trialists' Collaborative Group (EBCTCG) (2005). Effects of radiotherapy and of differences in the extent of surgery for early breast cancer on local recurrence and 15-year survival: an overview of the randomised trials. Lancet.

[22] Voogd AC, Nielsen M, Peterse JL, Blichert-Toft M, Bartelink H, Overgaard M, van Tienhoven G, Andersen KW, Sylvester RJ, van Dongen JA; Danish Breast Cancer Cooperative Group. Breast Cancer Cooperative Group of the European Organization for Research and Treatment of Cancer (2001). Differences in risk factors for local and distant recurrence after breast-conserving therapy or mastectomy for stage I and II breast cancer: pooled results of two large European randomized trials. J Clin Oncol.

[23] van der Sangen MJ, van de Wiel FM, Poortmans PM, Tjan-Heijnen VC, Nieuwenhuijzen GA, Roumen RM, Ernst MF, Tutein Nolthenius-Puylaert MC, Voogd AC (2011). Are breast conservation and mastectomy equally effective in the treatment of young women with early breast cancer? Long-term results of a population-based cohort of 1,451 patients aged ≤ 40 years. Breast Cancer Res Treat.

[24] van der Leest M, Evers L, van der Sangen MJ, Poortmans PM, van de Poll-Franse LV, Vulto AJ, Nieuwenhuijzen GA, Brenninkmeijer SJ, Creemers GJ, Voogd AC (2007). The safety of breast-conserving therapy in patients with breast cancer aged ≤40 years. Cancer.

[25] Cao JQ, Olson RA, Tyldesley SK (2013). Comparison of recurrence and survival rates after breast-conserving therapy and mastectomy in young women with breast cancer. Curr Oncol.

[26] Mahmood U, Morris C, Neuner G, Koshy M, Kesmodel S, Buras R, Chumsri S, Bao T, Tkaczuk K, Feigenberg S (2012). Similar survival with breast conservation therapy or mastectomy in the management of young women with early-stage breast cancer. Int J Radiat Oncol Biol Phys.

[27] Chan A, Pintilie M, Vallis K, Girourd C, Goss P (2000). Breast cancer in women ≤35 years: review of 1002 cases from a single institution. Ann Oncol.

[28] Bantema-Joppe EJ, van den Heuvel ER, de Munck L, de Bock GH, Smit WG, Timmer PR, Dolsma WV, Jansen L, Schröder CP, Siesling S, Langendijk JA, Maduro JH (2013). Impact of primary local treatment on the development of distant metastases or death through locoregional recurrence in young breast cancer patients. Breast Cancer Res Treat.

[29] Weissenbacher TM, Zschage M, Janni W, Jeschke U, Dimpfl T, Mayr D, Rack B, Schindlbeck C, Friese K, Dian D (2010). Multicentric and multifocal versus unifocal breast cancer: is the tumor-node-metastasis classification justified?. Breast Cancer Res Treat.

[30] Cancello G, Maisonneuve P, Rotmensz N, Viale G, Mastropasqua MG, Pruneri G, Montagna E, Iorfida M, Mazza M, Balduzzi A, Veronesi P, Luini A, Intra M, Goldhirsch A, Colleoni M (2013). Progesterone receptor loss identifies Luminal B breast cancer subgroups at higher risk of relapse. Ann Oncol.

